# Socioeconomic deprivation and its association with polypharmacy in England: results from a national cross-sectional survey

**DOI:** 10.1007/s11096-025-01990-4

**Published:** 2025-08-23

**Authors:** Yusuff Adebayo Adebisi, Najim Z. Alshahrani, Duaa Abdullah Bafail

**Affiliations:** 1 College of Social Sciences, University of Glasgow, Glasgow, UK; 2 Department of Family and Community Medicine, Faculty of Medicine, University of Jeddah, Jeddah, Saudi Arabia; 3 Department of Clinical Pharmacology, Faculty of Medicine, King Abdulaziz University, Jeddah, Saudi Arabia

**Keywords:** Polypharmacy, Socioeconomic deprivation, Multimorbidity, Public health, England

## Abstract

**Introduction:**

Polypharmacy is a growing public health concern, yet its association with area-level socioeconomic deprivation in England has been under-explored.

**Aim:**

To investigate whether socioeconomic deprivation, measured by the Index of Multiple Deprivation (IMD), is associated with polypharmacy among adults.

**Method:**

We analysed cross-sectional data from the 2021 Health Survey for England, including 1705 adults aged 16+ who completed nurse visits and reported prescribed medication use in the past week. Polypharmacy was defined as the use of five or more prescribed medications. IMD scores were categorised into quintiles (least to most deprived). Multivariable logistic regression estimated adjusted odds ratios (ORs) with 95% confidence intervals (CIs), controlling for age, sex, ethnicity, multimorbidity, obesity, smoking, alcohol use, and GP visit frequency. A polynomial contrast test assessed linear trends, and adjusted predicted probabilities were calculated to illustrate the deprivation–polypharmacy gradient.

**Results:**

In the fully adjusted model, adults residing in the most deprived IMD quintile had significantly higher odds of polypharmacy (OR 1.82; 95% CI 1.09–3.04; *p* = 0.022) compared to those living in the least deprived areas. No statistically significant associations were observed for intermediate quintiles. A polynomial contrast test confirmed a significant linear trend across IMD levels (*p* = 0.010), indicating that the odds of polypharmacy increased progressively with greater area-level deprivation. This gradient was further illustrated by adjusted predicted probabilities, which rose from 18.3% (95% CI 15.3–21.3%) in the least deprived quintile to 24.6% (95% CI 20.1–29.2%) in the most deprived (*p* < 0.001).

**Conclusion:**

Socioeconomic deprivation is independently associated with polypharmacy, even after adjusting for multimorbidity and other confounders, highlighting persistent health inequalities within England’s healthcare system. Targeted strategies, including regular medication reviews and enhanced access to care in deprived communities, may help mitigate risks and promote equity in prescribing practices.

## Impact statements

- This study demonstrates that area-level socioeconomic deprivation is a key driver of polypharmacy, even in a universal healthcare system, suggesting that equal access alone does not eliminate treatment-related inequalities.
- Findings support the global relevance of integrating area-based deprivation metrics into clinical risk assessment and medication review strategies to improve prescribing equity.
- Health systems worldwide facing rising multimorbidity can use these insights to design targeted, socially responsive interventions that reduce the burden of inappropriate or excessive medication use.
- Clinicians should consider the influence of socioeconomic context, alongside clinical factors, when reviewing medications, as patients from more deprived areas may be at higher risk of polypharmacy-related harm despite similar health profiles.

## Introduction

Polypharmacy, commonly defined as the concurrent use of five or more medications, has become a growing public health concern, particularly in aging populations and among individuals with multiple chronic conditions [1–3]. In England, the prevalence of polypharmacy has risen significantly over the past two decades, driven by an increase in multimorbidity, improved access to healthcare, and the availability of pharmacological treatments [4, 5]. While polypharmacy can be beneficial in managing complex health needs, it is also associated with adverse outcomes, including increased risk of drug interactions, reduced medication adherence, and higher healthcare costs [1, 6]. A recent report by the Department of Health and Social Care estimated that polypharmacy affects over 15% of adults in England, with the highest rates observed among older adults and those with chronic diseases such as diabetes and cardiovascular conditions [7]. Understanding the determinants of polypharmacy is critical to developing strategies that optimize medication use and mitigate its potential harms.

Socioeconomic deprivation, a key determinant of health, has been increasingly recognized as a factor influencing health outcomes and healthcare utilization [8]. In England, the Index of Multiple Deprivation (IMD) is widely used to measure deprivation across domains such as income, education, employment, and access to services, categorizing areas into quintiles from least to most deprived [9]. Research has consistently shown that individuals living in socioeconomically deprived areas experience higher rates of chronic illness, poorer access to preventive care, and greater reliance on pharmacological interventions [10, 11]. For example, a recent study found that individuals in the most deprived quintiles were more likely to have multimorbidity compared to those in the least deprived quintiles [12], potentially leading to increased medication use.

Internationally, a growing body of literature has examined the link between socioeconomic status (SES) and polypharmacy. A recent systematic review and meta-analysis synthesized findings from 54 studies and concluded that lower SES was consistently associated with higher odds of polypharmacy among older adults [3]. Most of these studies measured SES using individual-level indicators such as education, income, or occupation. Only one study in the review used an area-level measure of deprivation—an important gap given that health services planning and policy interventions often rely on area-based indicators [3]. Area-level measures, such as the IMD, are key for identifying geographic patterns of inequality and for targeting resources and interventions at the community or population level, especially where individual-level data may be unavailable or impractical to collect. Recent research in England has largely reinforced this focus on individual SES. For example, the Cognitive Function and Ageing Studies (CFAS I and II) examined the association between educational attainment and polypharmacy among adults aged 65 and over in England, finding that fewer years of education were associated with higher odds of polypharmacy [4]. While this contributes further valuable insight into the role of individual-level socioeconomic indicators, the relationship between area-based deprivation and polypharmacy within England's healthcare system remains underexplored. In addition, existing studies have largely focused on older adults [3], whereas our study includes all adults aged 16 years and over, allowing for a broader examination of socioeconomic inequalities in medication use across the adult life course.

### Aim

This study examined the association between socioeconomic deprivation, measured using IMD quintiles, and polypharmacy among adults in England. The primary objective was to determine whether individuals living in more deprived areas were more likely to experience polypharmacy, and if so, to quantify the strength of this association.

## Method

### Study design and data source

This study utilized a cross-sectional design to investigate the association between socioeconomic deprivation and polypharmacy among adults in England, using data from the Health Survey for England (HSE) 2021. The HSE is an annual survey commissioned by NHS England (formerly NHS Digital), designed to provide nationally representative data on the health and lifestyles of individuals living in private households in England [13]. The 2021 survey, conducted by the National Centre for Social Research (NatCen) and University College London (UCL), adapted its methodology due to the COVID-19 pandemic, shifting from face-to-face interviews to remote telephone and video interviews, with nurse visits resuming in October 2021 for a subset of participants [13].

The HSE 2021 employed a stratified random probability sample of households, selecting 12,798 addresses across 711 postcode sectors, comprising 9774 addresses in 543 postcode sectors for the core sample and 3,024 addresses in 168 postcode sectors for the reserve sample [13]. Fieldwork was conducted from January 2021 to June 2022, achieving a household response rate of 32%. In total, 5880 adults aged 16 and over and 1240 children aged 0 to 15 were interviewed, with 1,705 adults and 250 children completing a nurse visit. Data collection involved a Stage 1 interview (via telephone or video) and, for 89% of participating households, a Stage 2 nurse visit, which included physical measurements and biological sample collection.

### Participant selection and study population

For this analysis, we focused on adults aged 16 years and older who completed the HSE 2021 nurse visit, as the nurse questionnaire included detailed questions on medication use necessary for defining polypharmacy. In 2021, not all households were eligible for the nurse visit due to adaptations in the survey methodology. In each primary sampling unit (PSU), 16 out of 18 addresses were selected at random in advance; within these households, all adults and children who were interviewed during the Stage 1 interview were eligible for the nurse visit. In the remaining two households per PSU, nurse visits were not offered. This process resulted in 1,705 adults completing the nurse visit, which is the final analytical sample. All participants were included regardless of multimorbidity status to allow for a comprehensive population-level analysis of polypharmacy. This approach enabled us to assess whether area-level socioeconomic deprivation is associated with increased medication burden independently of diagnosed chronic conditions.

### Variables

#### Outcome: polypharmacy

Polypharmacy was defined as the concurrent use of five or more prescribed medications within the past seven days, a threshold commonly used in the literature to indicate increased risk of adverse drug interactions and healthcare burden [1–3]. Medication use was assessed based on participant self-report of prescribed treatments taken in the previous week, excluding contraceptives and nicotine-dependence therapies in accordance with standard practice. Responses were grouped into nine categories representing the number of medications taken, from none to eight or more. Medications covered a wide range of treatments for diverse conditions, including cardiovascular drugs (e.g. diuretics, ACE inhibitors), lipid-lowering agents, antidiabetic medications (e.g. metformin), respiratory treatments for asthma and chronic obstructive pulmonary disease (COPD), mental health medications (e.g. antidepressants, antipsychotics, and hypnotics), proton pump inhibitors (PPIs), non-steroidal anti-inflammatory drugs (NSAIDs), analgesics, and antibacterials. For analysis, a binary outcome variable was created: individuals reporting the use of five or more prescribed medications were classified as having polypharmacy (coded as 1), while those reporting fewer than five were classified as not having polypharmacy (coded as 0).

#### Exposure: socioeconomic deprivation

The primary exposure was socioeconomic deprivation, measured using the Index of Multiple Deprivation (IMD) 2019, a composite measure of deprivation across seven domains: income, employment, education, health, crime, barriers to housing and services, and living environment [14]. IMD scores were assigned to participants based on their residential postcode and categorized into quintiles, with Quintile 1 representing the least deprived areas and Quintile 5 the most deprived. The IMD was chosen as the exposure variable because it provides a robust, area-based measure of socioeconomic deprivation, widely used in UK health research to assess social inequalities [14].

#### Covariates

We adjusted for several covariates known to influence medication use and deprivation, collected during the Stage 1 interview and nurse visit. These included age (categorized as 16–44, 45–54, 55–64, and 65 + years), sex (male/female), and ethnicity (categorized as White, Non-White, and Others, based on self-reported ethnic origin, with Non-White including Black, Asian, and Mixed categories). Clinical factors included multimorbidity (defined as the presence of two or more doctor-diagnosed conditions such as hypertension, diabetes, or raised cholesterol, as reported in the nurse interview, and categorized as none, 1 condition, 2–3 conditions, or 4 + conditions) and obesity status (categorized as Obese, Not Obese, or missing, based on body mass index measurements taken during the nurse visit). Lifestyle factors comprised cigarette smoking status (current smoker, ex-regular smoker, never regular smoker) and alcohol consumption (categorized as non-drinker in the last 12 months, low-risk drinker [up to 14 units per week], higher-risk drinker [> 14 units per week], or missing, based on self-reported drinking behaviour). Healthcare access was assessed by the frequency of GP visits in the past 12 months (categorized as 0, 1 or 2, 3 to 5, or 6 or more visits), derived from general health questions asked of all participants during the Stage 1 interview. These covariates were selected based on their established associations with polypharmacy and deprivation in prior studies [15–18]. Missing and unreported values in the covariates were classified in a separate category.

### Statistical analysis

Descriptive statistics were used to summarize the characteristics of the study population, stratified by polypharmacy status, and presented as unweighted frequencies and percentages. Chi-square tests were used to examine differences between categorical variables across polypharmacy status. Fisher’s exact test was applied where expected cell counts were less than 5, ensuring accurate statistical inference in small sample sizes. To estimate the association between socioeconomic deprivation and polypharmacy, we employed multivariable logistic regression, modelling polypharmacy as a binary outcome, with IMD quintiles as the primary exposure (reference: Quintile 1, least deprived). The model adjusted for age, sex, ethnicity, multimorbidity, obesity, smoking status, alcohol consumption, and GP visit frequency to control for potential confounding. Odds ratios (ORs) with 95% confidence intervals (CIs) were reported to quantify the strength of the association.

A polynomial contrast test was used to assess the trend in the association between deprivation and polypharmacy, testing for a linear relationship across deprivation quintiles. To illustrate the relationship graphically, we computed adjusted predicted probabilities of polypharmacy across IMD quintiles, accounting for all covariates in the model. Statistical significance was set at *p* < 0.05. To assess the robustness of our findings, we conducted sensitivity analyses using alternative polypharmacy definitions: ≥ 4 medications and a stricter ≥ 6 medications within the past seven days. The logistic regression model was repeated with these modified thresholds to determine if the association between deprivation and polypharmacy persisted. All regression analyses incorporated survey weights to account for unequal selection probabilities, non-response, and ensure representativeness of the English adult population [13]. All analyses were conducted using Stata (version 18).

### Ethics approval

This study is a secondary analysis of Health Survey for England (HSE) 2021 data, so ethical approval was not sought. The original HSE 2021 study received approval from the East Midlands Nottingham 2 Research Ethics Committee (Reference no. 15/EM/0254), ensuring the protection of participants’ rights, safety, and wellbeing.

## Results

Of the 1,705 participants (no polypharmacy: n = 1,355; polypharmacy: n = 350), polypharmacy was significantly associated with older age (χ^2^ = 199.8, *p* < 0.001), with 71.4% aged 65 + years compared to 32.0% in the no-polypharmacy group. Multimorbidity (χ^2^ = 415.4, *p* < 0.001) was more common in the polypharmacy group, with 14.6% having four or more conditions compared to 1.4% in the no-polypharmacy group. Sex differences were marginal (χ^2^ = 3.2, *p* = 0.075), with 49.1% of the polypharmacy group being male versus 43.8% in the no-polypharmacy group. Ethnicity differed significantly (χ^2^ = 10.3, *p* = 0.004), with 95.1% of the polypharmacy group identifying as White, compared to 89.7% in the no-polypharmacy group.

Differences were also observed in obesity (χ^2^ = 43.6, *p* < 0.001), with a lower proportion of obese individuals in the polypharmacy group (62.9%) than in the no-polypharmacy group (77.4%). Ex-regular smoking (χ^2^ = 33.8, *p* < 0.001) and non-drinking (χ^2^ = 33.4, *p* < 0.001) were more common among those with polypharmacy. GP consultation frequency (χ^2^ = 134.1, *p* < 0.001) also differed, with 24.2% of the polypharmacy group reporting six or more visits compared to 7.3% in the no-polypharmacy group. Socioeconomic deprivation (χ^2^ = 12.3, *p* = 0.015) was associated with polypharmacy, with a higher proportion of individuals in the most deprived quintile in the polypharmacy group (16.6%) compared to the no-polypharmacy group (11.8%), while the least deprived quintile had more individuals in the no-polypharmacy group (27.8%) than in the polypharmacy group (21.4%) (See Table 1).

**Table 1:** Participants’ characteristics by polypharmacy status

Characteristics	No polypharmacy (n = 1,355)	Polypharmacy (n = 350)	Overall (n = 1,705)	χ^2^, *p*-value
*Age group, n (%)*				χ^2^ = 199.8, *p* < 0.001
16–44	399 (29.4)	20 (5.7)	419 (24.6)	
45–54	238 (17.6)	20 (5.7)	258 (15.1)	
55–64	285 (21.0)	60 (17.2)	345 (20.2)	
65+	433 (32.0)	250 (71.4)	683 (40.1)	
*Sex, n (%)*				χ^2^ = 3.2, *p* = 0.075
Male	594 (43.8)	172 (49.1)	766 (44.9)	
Female	761 (56.2)	178 (50.9)	939 (55.1)	
*Ethnic Group, n (%)*				χ^2^ = 10.3, *p* = 0.004
White	1,216 (89.7)	333 (95.1)	1,549 (90.9)	
Non-White	109 (8.0)	15(4.3)	124 (7.2)	
Others	30 (2.3)	2 (0.6)	32 (1.9)	
*Morbidity/Multimorbidity, n (%)*				χ^2^ = 415.4, *p* < 0.001
None	783 (57.8)	50 (14.3)	833 (48.9)	
1 condition	365 (26.9)	68 (19.4)	433 (25.4)	
2–3 conditions	188 (13.9)	181 (51.7)	369 (21.6)	
4 + conditions	19 (1.4)	51 (14.6)	70 (4.1)	
*Obesity Status, n (%)*				χ^2^ = 43.6, *p* < 0.001
Obese	1,049 (77.4)	220 (62.9)	1,269 (74.4)	
Not Obese	236 (17.4)	117 (33.4)	353 (20.7)	
Missing	70 (5.2)	13 (3.7)	83 (4.9)	
*Cigarette Smoking, n (%)*				χ^2^ = 33.8, *p* < 0.001
Current smoker	135 (9.9)	27 (7.7)	162 (9.5)	
Ex-regular smoker	344 (25.4)	144 (41.1)	488 (28.6)	
Never regular smoker	876 (64.7)	179 (51.2)	1,055 (61.9)	
*Alcohol consumption, n (%)*				χ^2^ = 33.4, *p* < 0.001
Non-drinker in the last 12 months	180 (13.3)	88 (25.1)	268 (15.7)	
Low-risk drinker (up to 14 units per week)	831 (61.3)	201 (57.4)	1,032 (60.5)	
Higher-risk drinker (> 14 units per week)	325 (24.0)	59 (16.9)	384 (22.5)	
Missing	19 (1.4)	2 (0.6)	21 (1.2)	
*Times consulted a GP in the last 12 months, n (%)*				χ^2^ = 134.1, *p* < 0.001
0	521 (38.5)	70 (20.0)	591 (34.7)	
1 or 2	536 (39.6)	101 (28.9)	637 (37.4)	
3 to 5	199 (14.7)	94 (26.9)	293 (17.1)	
6 or more	99 (7.3)	85 (24.2)	184 (10.8)	
*IMD Quintile, n (%)*				χ^2^ = 12.3, *p* = 0.015
Least deprived	376 (27.8)	75 (21.4)	451 (26.5)	
2	332 (24.5)	74 (21.1)	406 (23.8)	
3	267 (19.7)	80 (22.9)	347 (20.3)	
4	220 (16.2)	63 (18.0)	283 (16.6)	
Most deprived	160 (11.8)	58 (16.6)	218 (12.8)	

In the crude model (Model 1), the odds of polypharmacy increased with greater deprivation. Compared with the least deprived quintile, significant associations were observed for Quintile 3 (OR 1.50, 95% CI 1.06–2.14, *p* = 0.023) and the most deprived quintile (OR 1.82, 95% CI 1.23–2.68, *p* = 0.003). The association for Quintile 4 was borderline and did not reach conventional significance (OR 1.44, 95% CI 0.99–2.09, *p* = 0.058). In the adjusted model, which controlled for age group, sex, ethnicity, multimorbidity, obesity status, cigarette smoking, alcohol consumption, and GP visit frequency, the odds ratios were attenuated. Compared with the least deprived group, there was no evidence of association for Quintile 2 (OR 1.00, 95% CI 0.64–1.56, *p* = 0.996), while associations for Quintile 3 (OR 1.49, 95% CI 0.95–2.33, *p* = 0.081) and Quintile 4 (OR 1.36, 95% CI 0.85–2.19, *p* = 0.200) did not reach statistical significance. However, the most deprived quintile remained significantly associated with higher odds of polypharmacy (OR 1.82, 95% CI 1.09–3.04, *p* = 0.022) (see Table 2).

**Table 2:** Crude and adjusted association between socioeconomic deprivation and polypharmacy

Model	Odds ratio (95% CI), *p*-value
*Model 1 (Unadjusted/Crude)*	
Least deprived	Reference
2	1.11 (0.78–1.59), *p* = 0.538
3	1.50 (1.06–2.14), *p* = 0.023
4	1.44 (0.99–2.09), *p* = 0.058
Most deprived	1.82 (1.23–2.68), *p* = 0.003
Final Model (Adjusted for age group, sex, ethnicity, morbidity/multimorbidity, obesity, cigarette smoking status, alcohol consumption, frequency of GP visits)	
Least deprived	Reference
2	1.00 (0.64–1.56), *p* = 0.996
3	1.49 (0.95–2.33), *p* = 0.081
4	1.36 (0.85–2.19), *p* = 0.200
Most deprived	1.82 (1.09–3.04), *p* = 0.022
IMD modeled as continuous variable (final model)	1.16 (1.04–1.30), *p* = 0.009
Polynomial contrast test for trend (final model)	Linear *p* = 0.010, Quadratic *p* = 0.892, Cubic *p* = 0.972, and Quartic *p* = 0.214

To assess whether polypharmacy increased progressively across deprivation levels, a polynomial contrast test for trend was conducted. The test confirmed a statistically significant linear trend (*p* = 0.010), indicating that the likelihood of polypharmacy increases progressively with greater socioeconomic deprivation. Higher-order contrasts (quadratic, cubic, and quartic) were non-significant, confirming that the relationship is best explained by a linear trend rather than a curvilinear pattern (See Table 2).

**Fig. 1:**
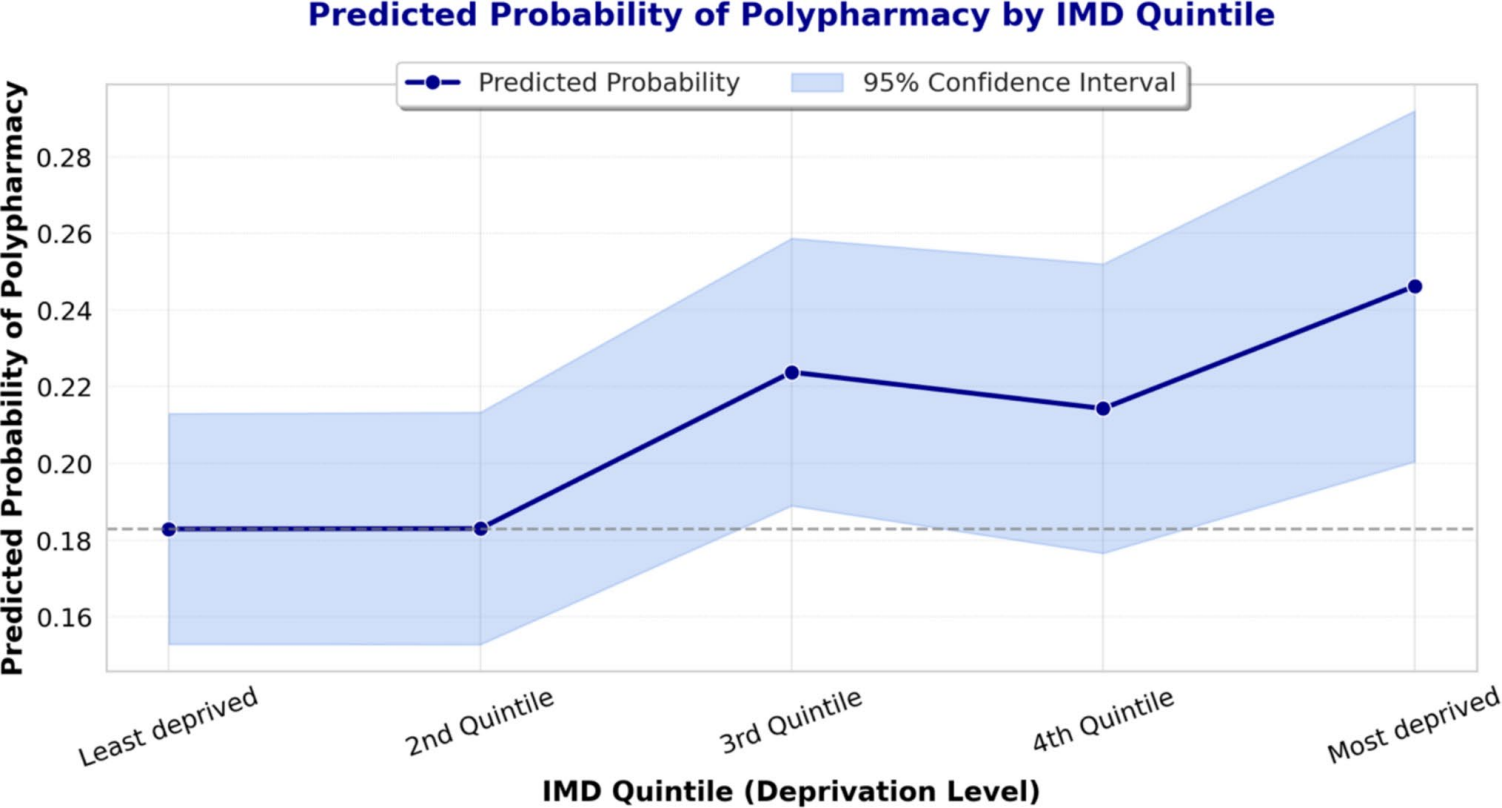
Adjusted predicted probability of polypharmacy by socioeconomic deprivation

The predicted probabilities, expressed as percentages, increase with deprivation level: least deprived (18.3%, 95% CI 15.3–21.3%), 2nd quintile (18.3%, 95% CI 15.3–21.3%), 3rd quintile (22.4%, 95% CI 18.9–25.9%), 4th quintile (21.4%, 95% CI 17.7–25.2%), and most deprived (24.6%, 95% CI 20.1–29.2%), all statistically significant (*p* < 0.001) (See Fig. 1).

In a sensitivity analysis defining polypharmacy as four or more medications, the association with socioeconomic deprivation remained significant, with the most deprived quintile showing an adjusted odds ratio of 1.65 (95% CI 1.02–2.68, *p* = 0.043) compared to the least deprived. For a stricter definition of six or more medications, the association also held, with the most deprived quintile having an adjusted odds ratio of 1.76 (95% CI 1.01–3.04, *p* = 0.044) compared to the least deprived, reinforcing the primary findings’ consistency across varying thresholds.

## Discussion

This study explored the relationship between socioeconomic deprivation, measured by the Index of Multiple Deprivation, and polypharmacy among adults in England, drawing on data from the Health Survey for England 2021. The adjusted logistic regression analysis revealed that individuals in the most deprived quintile faced 82% higher odds of polypharmacy compared to those in the least deprived quintile, even after controlling for variables such as age, sex, ethnicity, multimorbidity, obesity status, smoking, alcohol consumption, and GP visit frequency. Additionally, the adjusted predicted probabilities showed a steady increase from 18.3% in the least deprived quintile to 24.6% in the most deprived quintile, with all estimates reaching statistical significance. These findings confirm that socioeconomic deprivation significantly predicts polypharmacy, independent of health conditions and lifestyle factors. This pattern highlights a persistent inequality in medication use within England’s universal healthcare system, suggesting that deprivation influences not only the prevalence of illness but also how it is managed pharmacologically. Such insights underline the need to address social determinants alongside clinical factors when tackling polypharmacy.

The results align with previous research while adding new dimensions to the understanding of polypharmacy and deprivation. A study in Scotland reported higher polypharmacy rates in deprived areas, linking this trend to elevated multimorbidity [19]. Similarly, a systematic review of 54 studies across different countries noted that lower socioeconomic status often correlates with increased medication use, though adjustments for chronic conditions sometimes weakened this association [3]. In contrast, our analysis shows that the link between deprivation and polypharmacy persists even after accounting for multimorbidity, particularly in the most deprived quintile. This suggests that additional factors, potentially including differences in disease severity, duration, health-seeking behaviour, or prescribing practices, may contribute to higher medication use in disadvantaged groups. The clear gradient in predicted probabilities further sets this study apart, offering a visual and quantitative demonstration of how risk escalates with deprivation. Despite the NHS providing universal access, our results echo broader evidence of enduring health inequalities in England [20–23]. Unlike studies focused solely on disease prevalence, this research isolates polypharmacy as a distinct outcome, revealing how socioeconomic context shapes treatment patterns in ways that equal healthcare access alone cannot fully mitigate.

Several mechanisms might explain why deprivation is associated with increased polypharmacy. Research indicates that deprived populations often face barriers to non-pharmacological interventions, such as physiotherapy or dietary counselling, which could lead to a greater reliance on medications [24]. Our adjustment for GP visit frequency indicated that more frequent healthcare interactions in deprived areas might result in additional prescriptions, possibly due to time constraints during consultations. Qualitative studies support this idea, showing that general practitioners in under-resourced communities often favour quick pharmacological solutions over time-intensive alternatives [25]. Beyond patient-level factors, systemic issues within the healthcare system could also play a role. For example, deprived areas might have fewer multidisciplinary teams or limited funding for preventive programs, pushing healthcare providers toward prescribing as a primary strategy. While our data cannot directly confirm these mechanisms, the robustness of the association after adjusting for confounders points to structural influences rather than individual behaviours alone. This perspective shifts the focus from patient characteristics to the broader healthcare environment, offering a more systemic lens on the problem. In addition, broader social determinants, such as housing instability, food insecurity, psychosocial stress, lower health literacy, and limited social support, are likely to influence both the onset and management of chronic conditions [26]. These factors may increase dependence on pharmacological treatments as a compensatory mechanism in contexts where behavioural or lifestyle modifications are less feasible. Including such dimensions in future research could offer a fuller understanding of how deprivation shapes treatment pathways.

The COVID-19 pandemic forms an important contextual backdrop to this study, as data collection for the Health Survey for England 2021 coincided with the public health crisis. The pandemic may have influenced patterns of prescribing and medication use in several ways. For instance, disruptions to routine services, increased reliance on remote consultations, and delayed access to non-pharmacological interventions may have led to greater dependence on medications, particularly in deprived areas. In addition, individuals with long-term conditions may have received longer prescriptions to reduce healthcare contact, or additional treatments related to COVID-19 management or complications. These system-wide shifts could have temporarily altered polypharmacy patterns across the population, possibly exacerbating existing inequalities. Although we adjusted for GP visit frequency as a proxy for healthcare access, unmeasured pandemic-specific changes, such as altered prescribing thresholds or patient behaviours, may still have contributed to the observed disparities. Future research using post-pandemic data will be important to assess whether the socioeconomic gradient in polypharmacy persists beyond the unique pressures of the COVID-19 era.

The clinical and policy implications of these findings are substantial and warrant careful consideration. Polypharmacy increases the risk of adverse drug events, reduces adherence, and drives up healthcare costs, burdens that appear to fall disproportionately on deprived populations [27, 28]. The elevated odds in the most deprived quintile, combined with the rising predicted probabilities, suggest that these groups are particularly vulnerable to such risks. To address this, targeted interventions could include regular medication reviews to assess necessity and safety, as well as deprescribing initiatives to reduce unnecessary prescriptions. Expanding access to multidisciplinary care teams in high-deprivation areas could also shift reliance away from medications toward holistic approaches. Current NHS guidelines emphasize polypharmacy management, but they rarely highlight socioeconomic disparities as a focus [7]. Our study fills this gap by recommending that deprivation levels, such as IMD quintiles, be integrated into clinical decision-making tools to identify at-risk patients. Implementing these strategies could not only improve patient outcomes but also reduce inequities, aligning with the NHS’s mission to provide equitable care across all communities.

Clinical pharmacists play a crucial role in mitigating the risks of polypharmacy, particularly in settings with high levels of socioeconomic deprivation. Evidence from across Europe supports the effectiveness of pharmacist-led interventions such as medication reconciliation, structured reviews, and deprescribing protocols in improving prescribing safety and continuity of care [29]. For example, Slovenia’s national programme for pharmacist-led medication reconciliation at hospital discharge has demonstrated improved transition of care and reduced medication-related harm [29]⁠. In mental health care, clinical pharmacy services have also been shown to enhance treatment safety and interprofessional collaboration [30]. Expanding the reach of such services in deprived communities could help reduce inappropriate medication burden while supporting person-centred prescribing practices. Integrating clinical pharmacists into primary care teams, especially in under-resourced areas, may be a feasible and cost-effective strategy to address the socioeconomic disparities in polypharmacy observed in this study.

This study offers several notable strengths while also acknowledging important limitations. The use of a nationally representative dataset, the Health Survey for England, ensures that the findings are generalisable to the wider English population, further strengthened by the application of survey weights to correct for non-response and selection bias. Adjustment for a comprehensive set of confounders improves the accuracy of the estimated association between deprivation and polypharmacy, while the Index of Multiple Deprivation provides a robust and multidimensional measure of socioeconomic status. The inclusion of predicted probabilities adds interpretive value by visually demonstrating the socioeconomic gradient in polypharmacy, a technique not commonly used in similar analyses.

Given the cross-sectional design, reverse causation cannot be fully excluded; individuals with complex health needs and polypharmacy may be more likely to reside in deprived areas as a result of socioeconomic drift. However, area-level deprivation, as measured by the IMD, is typically stable and long-standing, lending support to the interpretation that deprivation more plausibly precedes and contributes to elevated polypharmacy risk. Additionally, the reliance on self-reported medication use introduces potential for recall bias, and the exclusion of over-the-counter medications may result in an underestimation of overall polypharmacy prevalence. Despite these limitations, which are common in related studies, the findings remain robust and provide meaningful insights into the socioeconomic patterning of polypharmacy.

## Conclusion

This study demonstrates that socioeconomic deprivation is a strong predictor of increased medication use in England. Even after accounting for multimorbidity and other influencing factors, individuals in the most deprived areas remain more likely to experience polypharmacy. In a healthcare system that aims to offer equal access to all, this pattern reveals persistent inequities that warrant urgent attention. To better understand the underlying causes, future research should adopt longitudinal approaches and explore the roles of prescribing habits and availability of non-medication-based care. In the meantime, targeted efforts in disadvantaged communities are essential to reduce the burden of excessive medication use and promote more equitable healthcare delivery.

## Data Availability

To download the dataset used in the analyses, please visit the https://ukdataservice.ac.uk/find-data/browse/health/.
